# Non‐native hosts of an invasive seaweed holobiont have more stable microbial communities compared to native hosts in response to thermal stress

**DOI:** 10.1002/ece3.9753

**Published:** 2023-01-24

**Authors:** Guido Bonthond, Anna‐Katrin Neu, Till Bayer, Stacy A. Krueger‐Hadfield, Sven Künzel, Florian Weinberger

**Affiliations:** ^1^ Institute for Chemistry and Biology of the Marine environment (ICBM) Carl‐von‐Ossietzky University Oldenburg Wilhelmshaven Germany; ^2^ GEOMAR Helmholtz Centre for Ocean Research Kiel Kiel Germany; ^3^ Department of Biology University of Alabama at Birmingham Birmingham Alabama USA; ^4^ Max Planck Institute for Evolutionary Biology Plön Germany

**Keywords:** Anna Karenina principle, beta diversity, common garden experiment, holobiont, invasive species, macroalgae, microbiota, stability

## Abstract

Seaweeds are colonized by a microbial community, which can be directly linked to their performance. This community is shaped by an interplay of stochastic and deterministic processes, including mechanisms which the holobiont host deploys to manipulate its associated microbiota. The Anna Karenina principle predicts that when a holobiont is exposed to suboptimal or stressful conditions, these host mechanisms may be compromised. This leads to a relative increase of stochastic processes that may potentially result in the succession of a microbial community harmful to the host. Based on this principle, we used the variability in microbial communities (i.e., beta diversity) as a proxy for stability within the invasive holobiont *Gracilaria vermiculophylla* during a simulated invasion in a common garden experiment. Independent of host range, host performance declined at elevated temperature (22°C) and disease incidence and beta diversity increased. Under thermally stressful conditions, beta diversity increased more in epibiota from native populations, suggesting that epibiota from non‐native holobionts are thermally more stable. This pattern reflects an increase in deterministic processes acting on epibiota associated with non‐native hosts, which in the setting of a common garden can be assumed to originate from the host itself. Therefore, these experimental data suggest that the invasion process may have selected for hosts better able to maintain stable microbiota during stress. Future studies are needed to identify the underlying host mechanisms.

## Graphical abstract

Seaweeds interact intimately with their associated microbial communities. Their capacity to manipulate microbiota may be important to their fitness in general. In the course of an invasion process, where an invader is faced with severe disturbance and novel conditions, there may be selective pressures for traits with which the host influences the associated microbiota. In this study, we simulated an invasion in a common garden and show that epibiota associated with an invasive seaweed are thermally more stable in populations with an invasion history compared with populations from the native range.

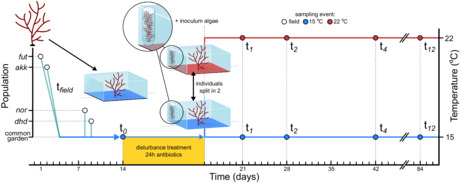

## 1 INTRODUCTION

Among the anthropogenic processes driving the contemporary loss of biodiversity and ecosystem functions, biological invasions play a major, but complicated role (Hooper et al., 2012). Their consequences for recipient ecosystems can range from devastating, to neutral, and even to positive, but they are generally difficult to predict as the effects are spread over multiple trophic levels (Simberloff, 2011). To become established in a new habitat, invaders have to face harsh conditions during transportation (e.g., unsuitable temperatures, anoxia, and darkness) followed by different and sometimes novel conditions in the non‐native environment. During and after transport, such conditions may act as selective filters that favor certain phenotypes (Bax et al., 2003; Blackburn et al., 2011). Compared with noninvasive populations or species, invaders tend to be more tolerant toward stressors (Walther et al., 2009), possess superior performance traits (e.g., growth rate, photosynthetic rate, and nitrogen use efficiency; Van Kleunen et al., 2010), and to have higher plasticity, which enables them to express more successful phenotypes across different environments (Richards et al., 2006). Moreover, invasive plants may be more *promiscuous* (or *flexible*) toward microbiota, which increases the probability of acquiring the same microbial functions from a different pool of potential symbionts available in the new environment (Klock et al., 2015; Maggia & Bousquet, 1994; Rodríguez‐Echeverría et al., 2011).

*Host promiscuity* (Perret et al., 2000) has also been termed *host generalism* (Rodríguez‐Echeverría et al., 2011) or *microbiome flexibility* (Voolstra & Ziegler, 2020) and was recently hypothesized to be a broadly occurring phenomenon among holobionts, which can promote the ability of hosts to respond and acclimate to environmental stress on short time scales (Voolstra & Ziegler, 2020). For invasive species which are transported to different environments, such a benefit would be particularly relevant as promiscuous hosts would not only be able to invade environments similar to the native environment, but would also be potentially invasive in a wider range of environments.

Whereas invasive plants have a major global impact terrestrially (Morales & Traveset, 2009; Pimentel et al., 2005), invasive macroalgae, or seaweeds, have caused analogous ecosystem effects in marine systems (Williams & Smith, 2007). As holobionts (see definition in Bordenstein & Theis, 2015), macroalgae are continuously interacting with microbial organisms from the water column, which colonize and penetrate their surfaces and tissues (Wahl et al., 2012). Some of these fouling microbes may be harmless, or protective (e.g., Li et al., 2021; Saha & Weinberger, 2019), involved in the regulation of host morphogenesis (Spoerner et al., 2012) or spore release (Weinberger et al., 2007), or facilitate the acquisition of nitrogen and/or vitamins (Croft et al., 2006; Gerard et al., 1990; Kazamia et al., 2012). Other taxa may represent a threat as opportunistic or specialized pathogens (Egan et al., 2014; Egan & Gardiner, 2016; Saha & Weinberger, 2019; Weinberger et al., 1994, 1997). Macroalgae manipulate the associated microbial community, for instance with specialized metabolites that target microbial fouling agents by interfering with *quorum sensing* (Harder et al., 2012). They also produce cue metabolites to attract and deter protective and harmful or (opportunistically) pathogenic symbionts (Kessler et al., 2018; Saha & Weinberger, 2019).

Recent evidence suggests that similar to plant invasions, *host promiscuity* may also be important in seaweed invasions. Using the invasive rhodophyte *Gracilaria vermiculophylla* (Ohmi) Papenfuss (synonym: *Agarophyton vermiculophyllum*), Bonthond et al. (2021) conducted a common garden experiment, wherein native and non‐native populations were subjected to a simulated introduction, experiencing disturbance followed by exposure to a new environment. Compared with native hosts, the epibiota of non‐native populations changed more with respect to their epibiota in the field. The epibiota of non‐native populations also became more similar to each other, suggesting that non‐natives are indeed more promiscuous toward potential symbionts and may therefore acclimate more easily to new conditions. The authors also observed that epibiota associated with native holobionts dispersed more within populations. The two measures of beta diversity between and within populations represent different characteristics of a community and especially in a common garden should be interpreted separately. Whereas the beta diversity between populations relates to the degree of change in epibiota in response to the environment, the *within‐population* beta diversity is instead related to the degree of stress experienced by holobionts from that population. This stress‐driven increase of variability in microbiota (or dispersion effect) is also known as the *Anna Karenina Principle* (Zaneveld et al., 2017), which predicts that microbiota disperse due to a relative increase in stochastic processes (or relative decrease in deterministic processes) acting on the holobiont. Therefore, the findings of Bonthond et al. (2021) do not only suggest that non‐native hosts are more promiscuous (beta diversity is lower between non‐native populations) but may also indicate that non‐native holobionts are less susceptible to stress or have more stable communities (beta diversity is lower within non‐native populations).

Based on this idea, we aimed to specifically compare how epibiota associated with native and non‐native *G. vermiculophylla* populations disperse in response to stress. A common garden experiment, similar to Bonthond et al. (2021), was conducted to simulate an invasion event by disturbing *G. vermiculophylla*'s prokaryote communities with antibiotics. We also included a temperature treatment, subjecting native and non‐native holobionts to optimal and (moderately) stressful thermal conditions. Here, we assumed that as subjects in the same common garden are exposed to the same environment, only processes originating from the host can vary. This implies that differences in beta diversity, which reflect differences in the ratio of deterministic and stochastic processes acting on the epibiota and therewith stability, originate from the host as well and are most likely explained by an increase or decrease in processes with which the host influences its epibiota. With two thermally different common gardens, we extended this idea to compare dispersion between two environments that only differed in temperature, expecting that non‐native holobionts are less susceptible to thermal stress and therefore disperse less under stressful conditions.

*Gracilaria vermiculophylla*, native to the northeast Pacific, has become a widespread, invasive species along the coasts of North America, northwestern Africa, and Europe (see Krueger‐Hadfield et al., 2017, 2021 and references therein). The rhodophyte is known to be chemically well equipped to manipulate microbiota (Saha et al., 2016; Saha & Weinberger, 2019), and multiple lines of evidence suggest that the interaction between host and microbes has played an important role in its successful invasion (Bonthond et al., 2020, 2021; Saha et al., 2016; Wang, Wang, et al., 2017; Wang, Weinberger, et al., 2017). Within native and non‐native habitats, *G. vermiculophylla* covers wide latitudinal ranges, which are highly variable in temperature (Sotka et al., 2018). Not surprisingly, it is tolerant to a relatively wide temperature range. *Gracilaria vermiculophylla* has been found to have a thermal growth optimum ranging from 15 to 25°C (Nejrup et al., 2013; Yokoya et al., 1999). At lower temperatures (8°C), the alga grows slowly, but can survive for months in dark and nutrient‐free conditions, which may explain its ability to survive long‐distance transportation (Nyberg & Wallentinus, 2009). However, qualitative observations (F. Weinberger et al., unpublished data) also suggested that the risk of developing disease symptoms increases at 20°C and higher.

We collected algal thalli from native and non‐native *G. vermiculophylla* populations and cultivated these in a common garden at an optimal growth temperature of 15°C and an elevated temperature of 22°C. We specifically tested the hypotheses that (i) *G. vermiculophylla* holobionts perform better at 15°C compared to 22°C (i.e., less disease symptoms and more growth) and (ii) epibiota have lower beta diversity within populations at 15°C compared to 22°C. In addition, we expected that (iii) non‐native algae perform better at 22°C and (iv) their epibiota have lower within‐population beta diversity at this elevated temperature.

## 2 MATERIALS AND METHODS

### 2.1 Sample collection

The experiment was conducted with individuals from two native and two non‐native populations. These populations were also visited as part of the global field survey conducted by Bonthond et al. (2020) and included Futatsuiwa (Japan; collected on September 14, 2016), Akkeshi (Japan; September 15, 2016), Nordstrand (Germany; September 20, 2016) and Kiel (Germany; September 21, 2016, see Table S1 for details). Individuals were sampled with gloves and with at least a meter distance in between (Krueger‐Hadfield et al., 2017), stored in separate plastic bags, and transported to the laboratory in coolers. Apart from Akkeshi, where single algae were too small to obtain enough tissue for all field and experiment samplings, 12 individuals were collected from each site. In Akkeshi, pseudo‐individuals were created by pooling a number of individuals from within 20 cm^2^ into a single bag and from this point, these algae were treated in the same manner as the separate individuals from other populations. Within 12 h of collection, the first sampling (timepoint: *t*_field_) of epiphytic communities was conducted. This was done in the same manner as in Bonthond et al. (2020). In brief, epiphytic communities were sampled by taking an apical fragment of approximately 1 g from each alga with sterilized forceps. The sample was transferred to 50 ml tubes containing 15 ± 1 glass beads (4 mm) and 7.5 ml autoclaved seawater made from standard sea salt and distilled water using the salinities measured at the collection site. After vigorous vortexing for 3 min, the supernatant was filtered through 0.2 μm PCTA filters and the procedure was repeated one time, using the same algal fragment. The filters were stored in 2 ml tubes at −20°C immediately, and those taken in Japan were transported on dry ice to Germany. The remaining thallus was used for the experiment. Thalli collected in Japan were stored at 4°C until they were transported in separate plastic bags to Germany. The transportation time (from refrigerator to aquaria) was <24 h.

### 2.2 Experimental design and disturbance treatment

**FIGURE 1:**
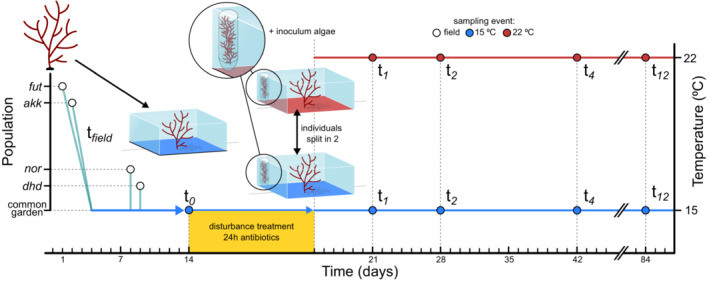
Schematic overview of the common garden experiment. The Japanese populations were collected on September 14 (Futatsuiwa; *fut*) and 15 (Akkeshi; *akk*) and arrived in the climate room on September 17. The populations from Germany were collected and brought to the climate room on September 20 (Nordstrand; *nor*) and September 21 (Kiel; *dhd*). The antibiotic treatment started on September 26 and lasted 24 h. Algae were subsequently separated into two parts into different tanks at 15°C and 22°C. These tanks also received the inoculum: a combination of living algal branches from all population in a 50 ml tube with openings, to function as a source of microbes. Epibiota were sampled in the field (*t*_field_), before the disturbance treatment (*t*_0_) and 1, 2, 4, and 12 weeks following the disturbance (*t*_1_, *t*_2_, *t*_4_, and *t*_12_, respectively).

Once in the climate‐controlled room, algal individuals were trimmed to 17 g wet weight and incubated in individual tanks containing artificial seawater (ASW; salinity: 24 g L^−1^), prepared from deionized water and sea salt, exposed to 12 h of light per day and bubbled with atmospheric air through an aeration stone inserted into the water with a tube. Five days after the last set of individuals arrived (from the population in Kiel), the sampling for the first timepoint was conducted (timepoint: *t*_0_) in the same way as samples were obtained in the field. Subsequently, the disturbance treatment was applied (see Figure 1 for a schematic overview of the sampling and experimental design). The treatment constituted a mixture of the antibiotics vancomycin (65 mg L^−1^) and cefotaxime (70 mg L^−1^) in a reduced water volume of 1 L. Assuming that a substantial part of microbiota was killed and the microbial community would be severely disturbed, the treatment was terminated after 24 hours, and the algae were rinsed with 1 L ASW. All 40 individuals and pseudo‐individuals were then split into two apical fragments of approximately 6 g wet weight and transferred into new separate 2 L tanks with 1.5 L ASW. For half of these thalli, the temperature was controlled at 15°C and the other half at 22°C, with both thermal levels containing one of the paired thalli originating from the same individual or pseudo‐individual. As the temperature in the climate room was 15°C, the 22°C treatment aquaria were placed in water‐filled basins containing heating elements. As a source of microbiota to reconfigure new microbial communities, each aquarium received an inoculum. This inoculum consisted of four 2 cm long branches (one for each population) taken from individuals that did not receive the antibiotic treatment. To prevent mixing with disturbed thalli, the inocula were not directly applied into the aquaria, but instead into 50 ml tubes, which contained openings on two sides, sealed with fine mesh (~1 mm) to prevent the exchange of algal fragments (see Figure 1). To promote the exchange of microbes, aeration stones were placed inside the tubes containing the inoculum.

The conditions in the climate room were kept constant, and epibiota were sampled from all algae after 1 week (*t*_1_), 2 weeks (*t*_2_), 4 weeks (*t*_4_), and 12 weeks (*t*_12_) by harvesting 1 g of tissue and executing the same vortexing method. To make the workload required for water exchanges and sampling events comprehensible, we conducted the experiment in a stacked order. Algae were divided into four groups, over which populations and treatments were balanced. Each group underwent the treatment and sampling with a 1‐day time lag. All samplings were conducted with sterilized equipment, and tanks were washed with bleach before use. Water was exchanged once per week.

### 2.3 Host performance

To obtain measures of host performance, we recorded the final wet weight at the end of the experiment (*t*_12_), summed it with the weight of the fragments harvested for the sampling of epibiota, divided this by the initial wet weight, and multiplied it by 100 to express relative growth rate (RGR) in average percentual growth per day. In addition, we measured the maximum quantum yield of photosystem II using a Diving‐PAM fluorometer (Heinz Walz GmbH, Effeltrich/Germany). For this purpose, algae were dark‐adapted for 15 min after the final sampling. During the experiment, we also observed the development of two types of disease or stress symptoms. The most prevalent of those symptoms was an increased brittleness of the thallus. We also observed thallus bleaching and decay (similar to Saha & Weinberger, 2019), which was often combined with fragmentation. Both thallus brittleness and decay were binary scored (0 or 1 for absence or presence of symptoms; see also Krueger‐Hadfield & Ryan, 2020; Sotka et al., 2018). To investigate correlations, we calculated Spearman rank coefficients among all four performance measures.

### 2.4 DNA extraction and amplicon sequencing

For the amplicon sequencing, we randomly selected 4 out of the 10 replicates in the experiment. The preserved filters, through which the suspension of epiphytes was filtered, were cut into fragments using sterilized scissors. Subsequently, DNA was extracted using the ZYMO Fecal/soil microbe kit (D6102; ZYMO Research, Irvine, CA, USA). Based on the two‐step PCR protocol from Gohl et al. (2016), we prepared 16S‐V4 amplicon libraries as described in Bonthond et al. (2020), using the KAPA HIFI HotStart polymerase (Roche, Basel, Switzerland) and the 16S‐V4 target and indexing primers (515F & 806R, Klindworth et al., 2013). All amplicons were pooled into two libraries, such that populations and treatments were balanced. We included four negative DNA extraction controls and four negative and positive PCR controls (mock communities; ZYMO D6311) in each library. After the final purification step, in which the pooled amplicons were run and re‐extracted from agarose gels, libraries were quantified using qPCR and sequenced at the Max Plank Institute for Evolutionary Biology (Plön, Germany) on the Illumina MiSeq platform as paired‐end 300‐bp reads. As most of the samples from the final timepoint (*t*_12_) yielded only a low number of reads, these samples were re‐amplified with 5 extra cycles in the first PCR and resequenced from a new amplicon library that was prepared following the same methods. Together with the original fastq files from the field samples that had been included in the study of Bonthond et al. (2020), files were demultiplexed allowing zero barcoding mismatches, assembled, quality filtered, and classified using the software Mothur v1.43.0 (Schloss et al., 2009) and the SILVA reference alignment v132 (Quast et al., 2013). After removing mitochondrial, chloroplast, eukaryotic, and unclassified sequences, the remaining sequences were clustered open reference to the OTUs from the field study in Bonthond et al. (2020), with the opticlust algorithm (Westcott & Schloss, 2017). To curate the final community matrix, we removed OTUs that were singleton in the full dataset, excluded samples with <1000 read counts, and deleted OTUs that had zero reads as a result of the preceding step. The demultiplexed sequences are deposited in the SRA database (accession: PRJNA564581, PRJNA612003, and PRJNA842363).

### 2.5 Statistical modeling

We used generalized linear mixed models (GLMMs) to analyze the effect of the temperature treatment on performance‐linked traits of the host and compare differences in performance between ranges. All traits were measured at the end of the experiment (*t*_12_) and therefore had, in contrast to the microbial count data, no repeated measures. The models were fitted as a function of *temperature*, *range*, their interaction, and the nested structure of *population identity* inside *range*. The random intercept *individual identity* was included in each model to represent the parent individual from which the thallus in the 15°C group and the thallus in the 22°C group originated. However, when individual identity did not explain variation, it was excluded and the model was run without random effects. We used a Gaussian distribution (with *identity* in the link function) to fit GLMMs on the RGR and the photosynthetic yield data. To satisfy the model assumptions, photosynthetic yield was rank transformed. The disease symptoms (thallus brittleness and tissue decay) were modeled using a binomial distribution with a logit in the link function.

Diversity was analyzed in terms of effective OTU numbers and evenness (measured as the probability of interspecific encounter, PIE), calculated with the R package mobr (McGlinn et al., 2019). GLMMs were then fitted on two subsets of the data. The first subset comprised the samples from the field (*t*_field_), before the disturbance treatment (*t*_0_) and the samples from the first timepoint (*t*_1_) after the disturbance treatment at 15°C. This model included *time* as factorial variable, *range*, the corresponding interaction, and *individual identity* nested in *population identity as* random intercepts. The second subset comprised all postdisturbance timepoints (*t*_1_, *t*_2_, *t*_4_, and *t*_12_) and was modeled as a function of *time*, *temperature*, *range*, the interactions, and *individual identity* nested in *population identity* as random intercepts. For the effective OTU numbers, the log‐transformed sequencing depth (LSD) was included as an offset.

We explored variation in community composition with nonmetric dimensional scaling (NMDS) on the full dataset (including all time points) and the postdisturbance dataset (*t*_1_, *t*_2_, *t*_4_, and *t*_12_), based on Bray–Curtis and Euclidean distances. Using the R package mvabund (Wang et al., 2012), multivariate generalized linear models (mGLMs) were fitted on the count matrix with the LSD as an offset prior to the scaling procedure to adjust for the effect of different read depths per sample. We then fitted mGLMs on the postdisturbance data, including different combinations of the variables time, temperature, and range to visualize their partial effects. All mGLMs assumed a negative binomial distribution with a natural logarithm in the link function. The NMDS was then conducted on the residuals of the models that were back‐transformed to the original scale. Group centroids and their 95% confidence regions were computed using the R package vegan (Oksanen et al., 2017). Statistics for the community response were obtained from the mGLM fitted on the postdisturbance dataset that included all variables (i.e., *time*, *temperature*, *range*, the corresponding interactions, and *population identity* nested in *range*), using the anova.manyglm function from the mvabund package, bootstrapping the univariate models with 500 iterations and individual identity as a blocking factor.

To analyze beta diversity within populations, we used Bray–Curtis and Euclidean distances calculated from the same community matrix as was used for the NMDS (i.e., adjusted for the effect of the sequencing depth). After computing the distances, we analyzed two parts of our dataset. We only considered distances between samples from the same range and the same temperature regimes. The first subset was prepared to analyze the effect of the disturbance on the beta diversity and included the predisturbance samples (*t*_field_, *t*_0_) and the first postdisturbance time point (*t*_1_) in the 15°C treatment. The GLMM used for the first model was a function of *time* as factor, *range*, and their interaction. The multilevel factors *population* and *individual combination* were included as random intercepts to account for dependency of distances calculated from the same individual pair. The postdisturbance subset of the data was used to analyze the effect of range and temperature on the microbiota dispersion. The GLMMs included the variables *time*, *temperature*, *range*, and all possible interactions.

### 2.6 OTUs related to host performance

OTUs of differential abundance between ranges and temperatures were identified from the univariate output of the mGLM fitted on postdisturbance time points. We considered OTUs with *p*‐values <.05 and with coefficients of which the 95% confidence limits were either both positive or both negative as differentially abundant. Following a joint modeling approach (Warton et al., 2015), we used the residuals of the mGLM to calculate Spearman correlations coefficients between OTUs in the samples of the final timepoint (*t*_12_) and the RGR and observed thallus brittleness disease symptom, for which 95% confidence intervals were obtained by bootstrapping with a 1000 iterations.

The univariate GLMMs for host performance traits and diversity measures were fitted using the R package lme4 (Bates et al., 2015). NMDS was conducted using the R package vegan (Oksanen et al., 2017). The mGLMs were run using the R package mvabund (Wang et al., 2012). To consider time in a flexible way in all postdisturbance models, we compared different versions of the full model based on the AICc or AICsum (for the mGLM), where *time* was specified as factorial and continuous variable (linear, log transformed, square root transformed, and as polynomial). Violations of model assumptions were verified visually with QQ plots and residual vs fitted plots for univariate and multivariate analyses.

## 3 RESULTS

The quality‐filtered OTU matrix counted 6,687,825 reads that were clustered into 14,209 OTUs, including 9746 reference OTUs from Bonthond et al. (2020) and 4463 new OTUs. Seven out of the 184 samples had less than 1000 reads after the quality filtering treatment and were excluded from downstream analyses (Table S1).

The most abundant phylum was Proteobacteria, followed by Bacteroidetes and Planctomycetes (Figure S1A). The abundance of Planctomycetes appeared to increase in the climate room (*t*_0_) and remained high with respect to abundances in the field until the last time point (*t*_12_) where their abundance was again lower. By contrast, Actinobacteria were of relatively high abundance in the field and nearly disappeared in the climate room but returned to high abundance levels again at the end of the experiment. The most abundant orders were Rhodobacterales, Flavobacteriales, and Alteromonadales (Figure S1B), and at the genus level, unclassified Rhodobacteraceae were the relative most abundant group (comprised of 529 OTUs), followed by the genera *Paraglaciecola* (23 OTUs), *Alteromonas* (3 OTUs), and *Granulosicoccus* (45 OTUs, Figure S1C). During the experiment, the latter three genera decreased in abundance at the end of the experiment. Instead, the Proteobacterial genus *Marivita* (27 OTUs) which was also more abundant in the field (*t*_field_) became one of the most abundant genera.

### 3.1 Host performance

**FIGURE 2:**
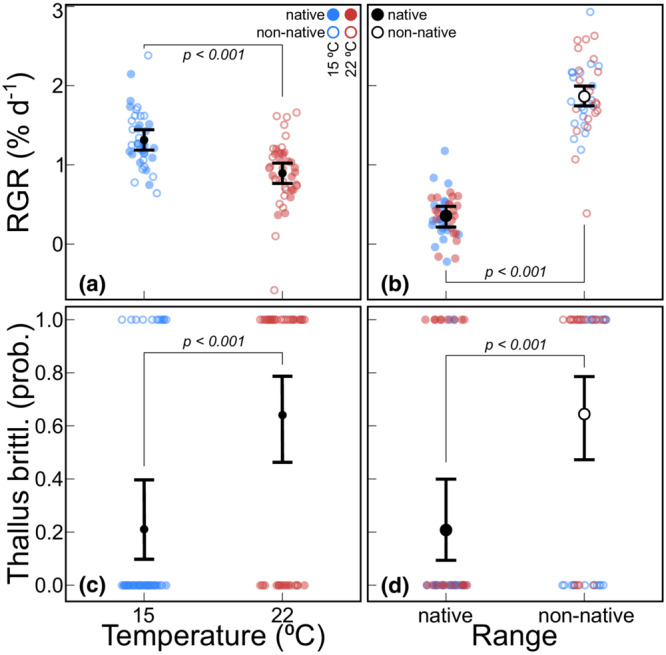
Marginal means of host performance traits recorded at the end of the experiment (*t*_12_) with 95% confidence intervals. The relative growth rate (RGR, a), measured as percentage wet weight increase per day between temperatures and between ranges (b). The probability of observing brittleness of the algal thallus between temperatures (c) and between ranges (d). RGR graphs include the partial residuals, and for thallus brittleness, the raw binary data were plotted.

The RGR was more than a third higher among algae at 15°C compared to 22°C and more than fivefold higher in non‐natives than in natives (*p* < .001, Figure 2a, b), but the interaction term was not significant (see Table S2a for all statistical output). Photosystem II quantum yields did not vary between temperatures or ranges. Of the observed disease symptoms, the thallus brittleness was more prevalent at 22°C and more common among non‐native algae (*p* < .001, Figure 2c, d) but did also not yield a significant interaction term. The observed thallus decay was overall low and did not vary with range or temperature. There were no strong correlations among the four response variables (maximum *ρ* = 0.31, between yield and thallus brittleness).

### 3.2 Alpha diversity

**FIGURE 3:**
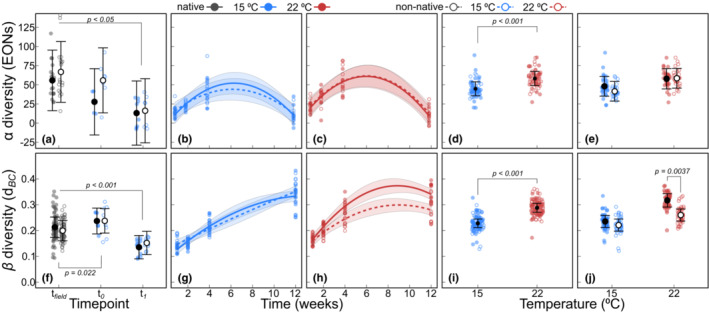
Marginal mean of effective OTU numbers (EON, panels a–e) and within‐population beta diversity measured as Bray–Curtis distances (f–j) with 95% confidence limits and partial residuals. Marginal means in the field (*t*_field_), before holobiont disturbance (*t*_0_) and 7 days after holobiont disturbance at 15°C (*t*_1_, a, f). Postdisturbance responses over time at 15°C (b, g) and 22°C (c, h), by temperature (d, i) and by range within temperature (e, j).

Effective OTU numbers and evenness did not differ between field (*t*_field_) and climate room before disturbance (*t*_0_), but there was a significant drop in both parameters upon the disturbance treatment, which was independent of the range (Figure 3a, Figure S2). For both effective OTU numbers and logit PIE, the models with time as a second‐order polynomial yielded the lowest AICc (Table S3). effective OTU numbers varied with time but not between ranges. Over the first three time points (*t*_1_, *t*_2_, and *t*_4_), effective OTU numbers increased but decreased again at the final time point (*t*_12_). Overall, effective OTU numbers were higher at 22°C and the interaction between time and temperature was also significant but the interaction between range and temperature was not (Figure 3b–e). Similarly, logit PIE varied with time and between temperatures, with high evenness at 22°C, but neither differed between ranges nor yielded significant interaction terms (Figure S2B–E, see Table S2b for all statistical output).

### 3.3 Within‐population beta diversity

**FIGURE 4:**
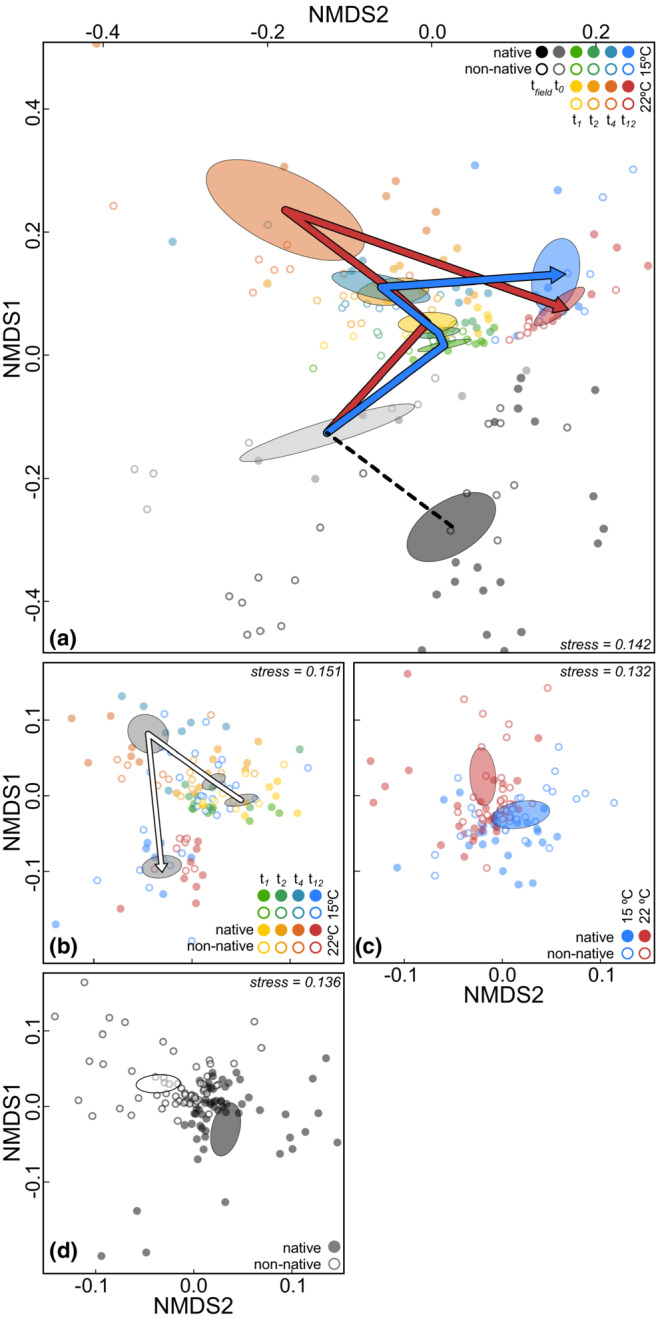
Nonmetric dimensional scaling (NMDS) based on the rescaled residuals from mGLMs including the LSD as offset to correct for the effect of sequencing depth using Bray–Curtis distances. Panel a includes samples from pre‐ and postdisturbance time points (*t*_field_, *t*_0_, *t*_1_, *t*_2_, *t*_4_, and *t*_12_). Blue and red arrows draw the postdisturbance trajectories of the 15 and 22°C temperature groups in time. The other panels show NMDS plots using rescaled residuals from mGLMs including all terms, except the variable of interest; time (b), temperature (c), and range (d). The 95% confidence regions of the group centroids are shown as ellipses. Note that some data points are outside the frame limits.

In general, NMDS based on Bray–Curtis and Euclidean distances revealed similar clustering patterns. The NMDS plots including pre‐ and postdisturbance data reflected a strong clustering by time (Figure 4a, b, Figure S2K, L). When corrected for the temporal effect, there were also clear effects of temperature (Figure 4c, Figure S2M) and range (Figure 4d, Figure S2N). These observations were also supported by the mGLM fitted on postdisturbance time points. Community composition differed with time, between temperatures, between ranges (*p* = .002), and also the interaction between temperature and range was significant (*p* = .012, Table S2c).

Beta diversity within populations based on Bray–Curtis and Euclidean distances revealed also similar patterns. Beta diversity increased from field to the climate room (*p*_Bray‐Curtis_ = .024, *p*_Euclidean_ = .004) but did not differ between ranges (*p* ≥ .525) and declined sharply following the disturbance treatment (*p* < .001, Figure 3f, Figure S2F). After the disturbance, within‐population beta diversity varied with time (*p* < .001) and followed an increasing trend (Figure 3g, h, Figure S2G, H) that was best fitted with a polynomial function (Table S3). Distances did not vary significantly between ranges (*p* ≥ .137). The main effect of temperature was highly significant for both distance measures, with substantially higher within‐population beta diversity at 22°C, compared to 15°C (*p* < .001, Figure 3i, Figure S2I). In addition, significant p‐values were found for the interactions between *range* and *time* (*p*_Bray‐Curtis_ = .015, *p*_Euclidean_ < .001), temperature and time (*p* < .001), and *range* and *temperature* (*p* ≤ .001). Post hoc comparisons indicated that the significant interaction between range and temperature was explained by higher beta diversity at 22°C in native populations compared with non‐natives °C(*p*_Bray‐Curtis_ = .004, *p*_Euclidean_ < .001), whereas natives and non‐natives did not differ in terms of within‐population beta diversity at 15°C (Figure 3j, Figure S2J, see Table S2d for all statistical output).

### 3.4 Host performance‐associated OTUs

In total, 382 OTUs were differentially abundant between temperature regimes, 206 of which specific to 15°C and 176 to 22°C (Figure S3A). Between ranges, we detected 578 differentially abundant OTUs, including 205 OTUs more abundant in native and 373 OTUs more abundant in non‐native populations (Figure S3B). Further, we found 29 OTUs to correlate positively and 9 OTUs to correlate negatively with RGR (Figure S3C) and 2 OTUs to correlate positively and 9 OTUs to correlate negatively with the thallus brittleness (Figure S3D).

## 4 DISCUSSION

### 4.1 Host performance declined with increased temperature (i)

In this simulated invasion event, where *G. vermiculophylla* holobionts were transported to a common garden and disturbed with antibiotics, we found strong differences in host performance between temperature regimes and between ranges. These differences were represented by RGR and a disease symptom that manifested itself as brittleness of the algal thallus. While the RGR was nearly a third lower at 22°C compared to 15°C (Figure 2a), the risk to develop thallus brittleness increased threefold (Figure 2c). These results are therefore in line with our first hypothesis, which posited that *G. vermiculophylla* performs better at 15°C compared to 22°C.

### 4.2 Epibiota dispersed more with increased temperature (ii)

As host performance declined with increased temperature, within‐population beta diversity increased. This observation is in line with the Anna Karenina principle, which predicts that conditions stressful to the host promote a decline in stability in the associated microbial community. It also supports our second hypothesis that thermal stress increases beta diversity in epibiota among individuals from the same population. Along with this increase in beta diversity, alpha diversity increased as well. This could be indirectly related to higher metabolic rates at elevated temperature (Clarke & Fraser, 2004; Gillooly et al., 2001), which may affect temperature‐dependent processes such as proliferation, dispersal, and succession. However, the increase in alpha diversity could at the same time be another indicator of a relaxation of influence from the host (and thus a relative increase in stochasticity) at elevated temperature. The production of defense‐related metabolites (Saha et al., 2016; Wang, Wang, et al., 2017) could, for instance, be reduced under stress, resulting in a higher probability for random microbes to temporarily settle in the community. On the contrary, increased alpha diversity may benefit the host under stressful conditions by promoting functional redundancy (Girvan et al., 2005) and support the holobiont to retain essential bacterial functions.

### 4.3 Host performance varies between ranges (iii)

We then hypothesized that at the elevated temperature (22°C), non‐native algae would show superior performance. This implies that, independent of a general effect of range, the interaction between range and temperature is significant. If non‐native algae are indeed more tolerant to thermal stress, performance should decline specifically for natives in the warm treatment, whereas performance among non‐natives would be affected less or not at all. Our results, however, do not support a significant interaction between *range* and *temperature*. Instead, they reflect a strong main effect of range on both RGR and thallus brittleness. While the RGR was over fivefold higher for non‐natives, which would suggest better performance, non‐natives were also threefold more likely to develop the thallus brittleness symptom (Figure 2c, d). Therefore, non‐native hosts do not simply perform better, but may be more susceptible to disease at the same time. Increased growth rates have been typically linked with invasions (Van Kleunen et al., 2010), and it has also been found that the selection for such invasiveness may come at the cost of other traits, such as for instance, traits linked to stress resistance or disease (Burns et al., 2007; Lambers & Poorter, 2004). However, Sotka et al. (2018) found in a common garden study that in response to heat (40°C), native *G. vermiculophylla* were more likely to bleach compared to non‐natives. In our experiment, the occurrence of bleaching was overall low and there was no detectable difference between natives and non‐natives. Possibly, bleaching is more likely to occur at higher temperatures than the treatment applied here, but the thallus brittleness we observed (and Sotka et al., 2018, did not) may be a symptom that is more specific to non‐native *G. vermiculophylla*. We note that Sotka et al. (2018) used apices in their experiments and may therefore have not been able to observe thallus brittleness.

### 4.4 Epibiota of native hosts disperse more with increased temperature (iv)

Finally, our results support the hypothesis that dispersion among native epibiota increases at elevated temperature. In addition to a general effect of temperature (Figure 3n, Figure S2D), within‐population dispersion at 22°C was indeed substantially higher in native populations than in non‐native populations (Figure 3o, Figure S2E). In contrast, at 15°C, dispersion was similar between the ranges. Therefore, our results show that under thermal stress, stochastic processes contributed relatively more to epibiota in natives than in non‐natives and this corroborates that microbial communities associated with non‐native hosts are more stable than those associated with native hosts. We note again that the algae from non‐native populations were cooled before transport and this could potentially have resulted in a slightly stronger disturbance compared with the treatment that native algae received. However, at *t*_0_ (after cooling and transport, before the antibiotic treatment), beta diversity did not differ between native and non‐native populations, which indicates that the observed differences in dispersion developed after the application of the treatment.

### 4.5 Within‐population beta diversity as a proxy for stability

Changes in beta diversity within a common garden may be informative in different ways. Whereas beta diversity among individuals from different populations (between‐population beta diversity) relates to changes within the holobiont with respect to the environment and other populations (microbiome flexibility; Voolstra & Ziegler, 2020 or host promiscuity; Bonthond et al., 2021), the within‐population beta diversity reflects the ratio between deterministic drivers (e.g., abiotic variables and mechanisms from the host) and stochastic drivers (e.g., historical contingency, mass effects, and microbe–microbe interactions) that act on the holobiont. Metabolites involved in attracting or repelling microbes (Saha & Weinberger, 2019), chemicals related to defense against fouling (Saha et al., 2016), for example, interfering with *quorum sensing* (Harder et al., 2012), traits related to morphology affecting the associated epibiota (Lemay et al., 2021), or promiscuity of the host toward potential symbionts (Bonthond et al., 2021; Klock et al., 2015) could represent important deterministic drivers that stabilize microbial communities. The Anna Karenina principle posits that as such host mechanisms are compromised or affected in response to stress, the associated microbial community is less shaped by these deterministic drivers and becomes more unstable (Zaneveld et al., 2017). Instead, stochastic processes acting on the microbial community become relatively more important, and consequently, beta diversity among replicated holobionts will increase.

Unlike in their natural habitat, where each population is exposed to a unique environment, in a common garden different populations are exposed to the same environment and therefore to the same environmental deterministic and stochastic processes. Turning the Anna Karenina principle around in the common garden, differences in within‐group beta diversity among groups of study could thus be used as a relative measure for the contribution of host mechanisms acting on the associated microbiota. Based on this idea, we used within‐population beta diversity as a proxy for stability. The present study provides an example of how different groups (here native and non‐native populations) can be compared.

### 4.6 More thermally stable holobionts may be more invasive

While we note that our experiment only included two native and two non‐native populations, these results support a difference in thermal stress tolerance between the distribution ranges and suggest that the degree of influence of the host on its epibiota may be higher among the non‐natives. Sotka et al. (2018) found evidence of increased heat tolerance and salinity stress tolerance in non‐native *G. vermiculophylla* in another common garden experiment and argued that the invasion process has selected for more stress‐tolerant genotypes. The higher epibiota dispersion in native populations we observed could indicate that such tolerance may be linked to how the host interacts with its epibiota. Especially in light of the invasion process, where the invader undergoes disturbance and must reassemble new harmless or beneficial epibiota while acclimating to a new environment, a selective pressure for mechanisms with which hosts influence epibiota could be beneficial. Ultimately, such adaptations would expand the range of environments where the host can successfully reassemble functional microbiota.

### 4.7 The role of host traits influencing epibiota in the invasion process

While the here observed beta diversity pattern is noteworthy, this study does not provide insight in putative traits that are differentially expressed in native and non‐native populations, which could explain differences in host influence. Further studies are required to better characterize the mechanisms by which *G. vermiculophylla* influences its epibiota and to identify whether these traits vary between the native and non‐native ranges. However, these data join the list of studies suggesting that changes in the interaction between host and microbiota in the *G. vermiculophylla* holobiont have played an important role in the invasion process (Bonthond et al., 2020, 2021; Saha et al., 2016; Saha & Weinberger, 2019; Wang, Wang, et al., 2017; Wang, Weinberger, et al., 2017). Most likely the observations made in these different studies have a common basis. For example, changes in traits related to defense against fouling (Saha et al., 2016) could promote promiscuity toward potential symbionts (Bonthond et al., 2021) and at the same time facilitate host influence under stressful conditions. Similarly, adaptations in traits related to the acquisition of vitamins (for some of which seaweeds are auxotroph; Croft et al., 2006; Kazamia et al., 2012) or other resources (e.g., Gerard et al., 1990) might enable the host to become more promiscuous toward symbionts that can potentially provide them and therewith indirectly support the synthesis of defense‐related metabolites, enhancing defense capacity in general and under stressful conditions. For now, we can only speculate and it remains a challenge for future experimental studies to identify the host mechanism(s) explaining host influence.

### 4.8 Host performance‐related OTUs

We detected some OTUs strongly correlated with host relative growth rate and thallus brittleness (Figure S3), which may represent taxa affecting host performance. For instance, OTU2 (classified to *Granulosicoccus*), which is an abundant core member of the *G. vermiculophylla* holobiont (Bonthond et al., 2020). Also in the present experiment, it was detected in high numbers as epiphyte and correlated positively with growth (Figure S3C). *Granulosicoccus* species have also been detected in high abundances (Ramirez‐Puebla et al., 2020) or as core microbiota (Park et al., 2022) on other seaweeds. OTU8 (classified to *Schizothrix*) is a core OTU (Bonthond et al., 2020) correlating negatively with thallus brittleness, which could hint at a protective role within the holobiont (Saha & Weinberger, 2019).

## 5 CONCLUSIONS

Based on the Anna Karenina principle, we used the variability of microbial communities (i.e., beta diversity) among replicated holobionts as a proxy for the ratio between stochastic and deterministic processes acting on the associated microbial community—and therewith as a proxy of stability within the holobiont. Using the invasive seaweed *G. vermiculophylla*, we simulated an invasion process in a common garden and demonstrated that epibiota beta diversity increases in response to thermal stress, exactly as the Anna Karenina principle predicts. Under stressed conditions non‐native populations—descending from algae that may have undergone selection as a result of the invasion—increased less in terms of beta diversity compared with native populations. We argue that these results imply that traits with which the host manipulates epibiota are less susceptible to thermal stress in non‐native populations and that the invasion process may have selected for this, resulting in non‐native populations that are more tolerant toward microbial pressures under a wider range of conditions.

## AUTHOR CONTRIBUTIONS

**Guido Bonthond:** Data curation (lead); formal analysis (lead); Investigation (equal); methodology (lead); validation (equal); writing – original draft (lead); writing – review and editing (lead). **Anna‐Katrin Neu:** Conceptualization (equal); investigation (lead); methodology (equal); resources (equal); writing – review and editing (equal). **Till Bayer:** Conceptualization (equal); funding acquisition (equal); investigation (equal); methodology (equal); project administration (equal); resources (equal); supervision (equal); validation (equal); writing – original draft (equal); writing – review and editing (equal). **Stacy A. Krueger‐Hadfield:** Data curation (equal); investigation (equal); methodology (equal); resources (equal); writing – review and editing (equal). **Sven Künzel:** Investigation (equal); resources (equal); writing – review and editing (equal). **Florian Weinberger:** Conceptualization (equal); funding acquisition (equal); data curation (equal); formal analysis (equal); investigation (equal); methodology (equal); project administration (equal); resources (equal); supervision (lead); validation (equal); writing – original draft (equal); writing – review and editing (equal).

## FUNDING INFORMATION

This study was funded by the German Research Foundation (DFG) grants awarded to FW (WE2700/5‐1) and TB (BA5508/2‐1) and start‐up funds from the College of Arts and Sciences at the University of Alabama at Birmingham to SAKH.

## Supporting information


Appendix S1

## Data Availability

The demultiplexed sequencing data generated for this study have been deposited in the Short Read Archive (SRA) under the bioproject accession numbers PRJNA564581, PRJNA612003, and PRJNA842363.
