# Supplementary material for: Non‐native hosts of an invasive seaweed holobiont have more stable microbial communities compared to native hosts in response to thermal stress
Source: Ecol Evol. 2023 Jan 24;13(1):e9753. doi: 10.1002/ece3.9753 (PMC9873590; doi:10.1002/ece3.9753)
Supplement: Supplementary file 1 — Appendix S1 [file ECE3-13-e9753-s001.pdf]

Supporting information

Non-native hosts of an invasive seaweed holobiont have more stable microbial communities compared to native hosts in response to thermal stress

Guido Bonthond[1,2,*], Anna-Katrin Neu[2], Till Bayer[2], Stacy A. Krueger-Hadfield[3], Sven Künzel[4], Florian Weinberger[2]

[1] Institute for Chemistry and Biology of the Marine environment (ICBM), Carl-von-Ossietzky University, Oldenburg, Schleusenstrasse 1, 26382 Wilhelmshaven, Germany
[2] GEOMAR Helmholtz Centre for Ocean Research Kiel, Düsternbrooker Weg 20, 24105, Kiel, Germany
[3] Department of Biology, University of Alabama at Birmingham, 1300 University Blvd, CH464, Birmingham, AL, 35294, USA
[4] Max Planck Institute for Evolutionary Biology, August-Thienemann-Straße 2, Plön, 24306, Germany
* corresponding author: guidobonthond@gmail.com

Table S1. Summary of samples remaining after data quality treatment and included in analyses

code	exp label (15/22)	stack group[1]	range	country	pop[2]	longitude (E)	latitude (N)	collection date	collectors[3]	included in analysis[4]					
										t_f	t_0	t_1	t_2	t_4	t_{12}
akk1	25/37	1	native	Japan	akk	144°56'59.30"	43°2'51.90"	15-9-16	FW,AN	x	x	15/22	15/22	15/22	15/22
akk2	26/38	1	native	Japan	akk	144°56'59.30"	43°2'51.90"	15-9-16	FW,AN	x					
akk3	27/39	1	native	Japan	akk	144°56'59.30"	43°2'51.90"	15-9-16	FW,AN	x	x	15/22	15/22	15/22	15/22
akk4	28/40	2	native	Japan	akk	144°56'59.30"	43°2'51.90"	15-9-16	FW,AN	x					
akk5	29/41	2	native	Japan	akk	144°56'59.30"	43°2'51.90"	15-9-16	FW,AN	x	x	15/22	15/22	15/22	15/22
akk6	30/42	3	native	Japan	akk	144°56'59.30"	43°2'51.90"	15-9-16	FW,AN	x					
akk7	31/43	3	native	Japan	akk	144°56'59.30"	43°2'51.90"	15-9-16	FW,AN	x	x	15/22	15/22	15/-	15/22
akk8	32/44	3	native	Japan	akk	144°56'59.30"	43°2'51.90"	15-9-16	FW,AN	x					
akk9	33/45	4	native	Japan	akk	144°56'59.30"	43°2'51.90"	15-9-16	FW,AN	x					
akk10	34/46	4	native	Japan	akk	144°56'59.30"	43°2'51.90"	15-9-16	FW,AN	x					
fut1	1/13	1	native	Japan	fut	144°15'28.40"	44°2'57.40"	14-9-16	FW,AN	x	x	15/22	15/22	15/22	15/22
fut2	2/14	1	native	Japan	fut	144°15'28.40"	44°2'57.40"	14-9-16	FW,AN	-					
fut3	3/15	1	native	Japan	fut	144°15'28.40"	44°2'57.40"	14-9-16	FW,AN	x	x	15/22	15/22	15/22	15/22
fut4	4/16	2	native	Japan	fut	144°15'28.40"	44°2'57.40"	14-9-16	FW,AN	x					
fut5	5/17	2	native	Japan	fut	144°15'28.40"	44°2'57.40"	14-9-16	FW,AN	x					
fut6	6/18	3	native	Japan	fut	144°15'28.40"	44°2'57.40"	14-9-16	FW,AN	x	-	15/22	15/22	15/22	15/22
fut7	7/19	3	native	Japan	fut	144°15'28.40"	44°2'57.40"	14-9-16	FW,AN	x					
fut8	8/20	3	native	Japan	fut	144°15'28.40"	44°2'57.40"	14-9-16	FW,AN	x	-	15/22	15/22	15/22	15/22
fut9	9/21	4	native	Japan	fut	144°15'28.40"	44°2'57.40"	14-9-16	FW,AN	x					
fut10	10/22	4	native	Japan	fut	144°15'28.40"	44°2'57.40"	14-9-16	FW,AN	x					
dhd1	37/85	1	nonnative	Germany	fdm	10°11'37.20"	54°21'49.80"	21-9-16	FW,AN	x	x	15/22	15/22	15/22	15/22
dhd2	38/86	1	nonnative	Germany	fdm	10°11'37.20"	54°21'49.80"	21-9-16	FW,AN	x					
dhd3	39/87	2	nonnative	Germany	fdm	10°11'37.20"	54°21'49.80"	21-9-16	FW,AN	-					
dhd4	40/88	2	nonnative	Germany	fdm	10°11'37.20"	54°21'49.80"	21-9-16	FW,AN	x					
dhd5	41/89	2	nonnative	Germany	fdm	10°11'37.20"	54°21'49.80"	21-9-16	FW,AN	x	x	15/22	15/22	15/22	15/22
dhd6	42/90	2	nonnative	Germany	fdm	10°11'37.20"	54°21'49.80"	21-9-16	FW,AN	x					
dhd7	43/91	3	nonnative	Germany	fdm	10°11'37.20"	54°21'49.80"	21-9-16	FW,AN	x	x	15/22	15/22	15/22	15/22
dhd8	44/92	3	nonnative	Germany	fdm	10°11'37.20"	54°21'49.80"	21-9-16	FW,AN	x					
dhd9	45/93	4	nonnative	Germany	fdm	10°11'37.20"	54°21'49.80"	21-9-16	FW,AN	x	x	15/22	15/22	15/22	15/22
dhd10	46/94	4	nonnative	Germany	fdm	10°11'37.20"	54°21'49.80"	21-9-16	FW,AN	x					
nor1	49/61	1	nonnative	Germany	nor	8°48'44.65"	54°29'9.34"	20-9-16	FW,AN	x	x	15/22	15/22	15/22	15/22
nor2	50/62	1	nonnative	Germany	nor	8°48'44.65"	54°29'9.34"	20-9-16	FW,AN	x					
nor3	51/63	2	nonnative	Germany	nor	8°48'44.65"	54°29'9.34"	20-9-16	FW,AN	x					
nor4	52/64	2	nonnative	Germany	nor	8°48'44.65"	54°29'9.34"	20-9-16	FW,AN	-					
nor5	53/65	2	nonnative	Germany	nor	8°48'44.65"	54°29'9.34"	20-9-16	FW,AN	x	x	15/22	15/22	15/22	15/22
nor6	54/66	2	nonnative	Germany	nor	8°48'44.65"	54°29'9.34"	20-9-16	FW,AN	x					
nor7	55/67	3	nonnative	Germany	nor	8°48'44.65"	54°29'9.34"	20-9-16	FW,AN	x	-	15/22	15/22	15/22	15/22
nor8	56/68	3	nonnative	Germany	nor	8°48'44.65"E	54°29'9.34"N	20-9-16	FW,AN	x					
nor9	57/69	4	nonnative	Germany	nor	8°48'44.65"E	54°29'9.34"N	20-9-16	FW,AN	x	x	15/22	15/22	15/22	15/22
nor10	58/70	4	nonnative	Germany	nor	8°48'44.65"E	54°29'9.34"N	20-9-16	FW,AN	x					

[1] *Stacking group to which samples in the experiment belonged*

[2] *Abbreviations for populations: Akkeshi (akk), futtasuiwa (fut), Kiel (dhd), Nordstrand (nor)*

[3] *Collectors: Florian Weinberger (FW), Anna-Katrin (AN)*

[4] *Crosses indicate field and t0 samples with more than a 1000 read counts after quality filtering included in the analyses. Samples taken during the experiment and passing the quality filtration steps are displayed as 15/22, corresponding to the temperature groups in Celsius. Dashes indicate samples not passing quality criteria whereas samples not included in the field study or experiment are represented by empty cells.*

Table S2. Complete statistical output of all models used in the analyses. **(A)** Analysis of deviance tables for host performance

		F	Df	Df.res	Pr(>F)[4]	
RGR[1]	temperature	21.5432304	1	38	**4.0416E-05**	***
	range	275.141554	1	36	**1.9328E-18**	***
	temperature :range	0.696	1	38	4.094E-01	
	range:pop	43.441	2	36	**2.528E-10**	***
		F	Df	Df.res	Pr(>F)	
rank(yield)[1]	temperature	0.50804403	1	38	0.48034104	
	range	3.31142796	1	36	0.07712345	.
	temperature:range	3.53111592	1	38	0.06791308	.
	range:pop	1.19242413	2	36	0.31518686	
		LR χ2	Df	Df.res	Pr(>Chisq)	
TB[2]	temperature	12.264	1.000	38	**0.00046166**	***
	range	11.774	1.000	36	**0.00060053**	***
	temperature:range	0.280	1.000	38	0.59690288	
	range:pop	3.968	2.000	36	0.137550	
		χ2	Df	Df.res	Pr(>Chisq)	
TD[3]	temperature	0.15895112	1	38	0.69012404	
	range	0.08753126	1	36	0.76733943	
	temperature:range	0.06817661	1	38	0.79401037	
	range:pop	0.2271278	2	36	0.89264715	

Abbreviations: Relative growth rate (RGR), Thallus brittleness (TB), Thallus decay (TD). Likelihood-ratio (LR), Degrees of Freedom (Df)

[1] Models assumed a Gaussian distribution and included 'individual identity' as random intercept. P-values were computed with type II Wald F-tests with Kenward-Roger Df

[2] The models assumed a binomial distribution with a logit in the link function. The random intercept 'individual identity' did not explain deviance and was exlcuded. P-values were computed with type II Likelihood-ratio tests.

[3] The models assumed a binomial distribution with a logit in the link function and included 'individual identity' as random intercept. P-values were computed with type II Wald χ^2 tests.

[4] Significance codes: p < 0.01 (.), p < 0.05 (*), p < 0.01 (**), p < 0.001 (***)

Table S2. Complete statistical output of all models used in the analyses. **(B)** Analysis of deviance tables and post-hoc pairwise comparisons for alpha diversity

				F	Df	Df.res	Pr(>F)[3]	
disturbance[1]	EON	timepoint		18.283	2.0	42.774	**1.826E-06**	***
		range		0.199	1.0	1.999	6.990E-01	
		timepoint:range		0.866	2.0	42.857	4.280E-01	
			**estimate**	**SE**	**Df**	**t-ratio**	**Pr(>t)**	
		t_{field} - t_0	11.2	12.1	54.5	0.927	0.6257	
		t_{field} - t_1	38.1	11.8	54.3	3.242	**5.700E-03**	**
		t_0 - $t_{1\text{-}15°C}$	26.9	9.7	34.1	2.788	**2.280E-02**	*
				**F**	**Df**	**Df.res**	**Pr(>F)**	
	PIE	timepoint		40.181	2.0	42.765	**1.512E-10**	***
		range		0.258	1.0	1.999	6.618E-01	
		timepoint:range		0.603	2.0	42.848	5.515E-01	
			**estimate**	**SE**	**Df**	**t-ratio**	**Pr(>t)**	
		t_{field} - t_0	0.296	0.174	47.8	1.694	0.2180	
		t_{field} - t_1	1.426	0.160	45.4	8.908	< 0.0001	***
		t_0 - $t_{1\text{-}15°C}$	1.130	0.200	35.0	5.664	< 0.0001	***
				**F**	**Df**	**Df.res**	**Pr(>F)**	
post-disturbance[2]	EON	poly(time, 2)		87.049	2.0	101.153	**< 2.22E-16**	***
		range		0.035	1.0	2.000	8.685E-01	
		treatment		13.269	1.0	101.126	**4.277E-04**	***
		poly(time, 2):range		0.541	2.0	101.167	5.840E-01	
		poly(time, 2):treatment		2.721	2.0	101.167	7.062E-02	
		range:treatment		0.115	1.0	101.134	7.355E-01	
		poly(time, 2):range:treatment		0.254	2.0	101.175	7.760E-01	
				**F**	**Df**	**Df.res**	**Pr(>F)**	
	PIE	poly(time, 2)		65.185	2.0	101.164	**6.477E-19**	***
		range		0.255	1.0	1.999	6.640E-01	
		temperature		8.503	1.0	101.135	**4.368E-03**	**
		poly(time, 2):range		0.103	2.0	101.179	9.024E-01	
		poly(time, 2):temperature		1.283	2.0	101.179	2.817E-01	
		range:temperature		0.249	1.0	101.144	6.189E-01	
		poly(time, 2):range:temperature		0.213	2.0	101.187	8.088E-01	

Abbreviations: Effective OTU Numbers (EON), Probability of interspecific encounter (PIE), Likelihood-ratio (LR), Degrees of Freedom (Df)

[1] Models assumed a Gaussian distribution and included 'population identity' and 'individual identity' as random intercepts. P-values were computed with type II Wald F-tests with Kenward-Roger Df.

[2] Tukey adjusted p-values for post-hoc timepoint comparisons were obtained using t-tests and Kenward-Roger Df, with timepoints mean estimates that were averaged over range.

[3] Significance codes: p < 0.01 (.), p < 0.05 (*), p < 0.01 (**), p < 0.001 (***)

Table S2. Complete statistical output of all models used in the analyses. **(C)** Analysis of deviance tables mGLMs

		Res.Df	Df.diff	Dev	Pr(>dev)[4]	
disturbance[1,3]		**Res.Df**	**Df.diff**	**Dev**	**Pr(>dev)**[4]	
	(Intercept)	65	NA	NA	NA	
	timepoint	63	2	16446.042	**0.002**	**
	range	62	1	11185.978	**0.002**	**
	timepoint:range	60	2	3520.131	**0.002**	**
post-disturbance[2,3]		**Res.Df**	**Df.diff**	**Dev**	**Pr(>dev)**	
	(Intercept)	126	NA	NA	NA	
	poly(time, 2)	124	2	35138.001	**0.002**	**
	temperature	123	1	5786.629	**0.002**	**
	range	122	1	10944.151	**0.002**	**
	temperature:range	121	1	2812.093	**0.012**	*

Abbreviations: Degrees of Freedom (Df)

[1] The mGLM was fitted on the subset of the data containing pre-disturbance timepoints t_{field}, t_0 and post-disturbance timepoint $t_{1\text{-}15°C}$.

[2] The mGLM was fitted on the subset of the data containing post-disturbance timepoints: t_1, t_2, t_4, t_{12}

[3] The models assumed a negative binomial distribution with a log in the link function and included the log-transformed sequencing depth as offset and 'individual identity' as blocking factor. P-values were computed by bootstrapping the univariate models with 500 iterations and Likelihood-ratio tests.

[4] Significance codes: p < 0.01 (.), p < 0.05 (*), p < 0.01 (**), p < 0.001 (***)

Table S2. Complete statistical output of all models used in the analyses. **(D)** Analysis of deviance and post-hoc comparison tables for beta-diversity within populations.

				estimate	F / SE	Df	Df.res / t-ratio	Pr	
disturbance[1]	Bray-Curtis	ANOVA			F	Df	Df.res	Pr(>F)[3]	
			timepoint		28.319	2.0	126.505	**6.863E-11**	***
			range		0.094	1.0	1.997	0.7879	
			timepoint:range		1.308	2.0	126.914	0.2741	
		PHPC[2]		estimate	SE	Df	t-ratio	Pr(>t)	
			t_{field} - t_0	-0.0310	0.01161	166.5	-2.673	**0.0224**	
			t_{field} - t_1	0.0626	0.00949	162.8	6.595	**< 0.0001**	***
			t_0 - $t_{1\text{-}15°C}$	0.0936	0.01401	80.3	6.681	**< 0.0001**	***
	Euclidean	ANOVA			F	Df	Df.res	Pr(>F)	
			timepoint		37.696	2.0	126.351	**1.450E-13**	***
			range		0.409	1.0	1.999	5.879E-01	
			timepoint:range		4.049	2.0	126.742	**1.974E-02**	*
		PHPC[2]		estimate	SE	Df	t-ratio	Pr(>t)	
			t_{field} - t_0	-9.18	2.84	166.1	-3.235	**4.200E-03**	**
			t_{field} - t_1	17.69	2.32	162.8	7.619	**< 0.0001**	***
			t_0 - $t_{1\text{-}15°C}$	26.87	3.43	80.1	7.841	**< 0.0001**	***
post-disturbance[1]	Bray-Curtis	ANOVA			F	Df	Df.res	Pr(>F)	
			poly(time,2)		400.145	2.0	155.318	**5.279E-62**	***
			range		1.692	1.0	1.997	0.323	
			temperature		21.138	1.0	155.274	**8.804E-06**	***
			poly(time,2):range		4.336	2.0	155.386	**0.015**	*
			poly(time,2):temperature		25.354	2.0	155.386	**2.955E-10**	***
			range:temperature		20.478	1.0	155.315	**1.193E-05**	***
			poly(time,2):range:temperature		2.575	2.0	155.428	0.079	.
		PHPC[3]		estimate	SE	Df	t-ratio	Pr(>t)	
			15°C: native - nonnative	0.0143	0.01680	20.3	0.852	0.4041	
			22°C: native - nonnative	0.0568	0.01770	24.6	3.202	**0.0037**	**
	Euclidean	ANOVA			F	Df	Df.res	Pr(>F)	
			poly(time,2)		681.316	2.0	155.659	**1.029E-77**	***
			range		5.841	1.0	1.991	1.375E-01	
			temperature		29.334	1.0	155.572	**2.270E-07**	***
			poly(time,2):range		7.977	2.0	155.793	**5.034E-04**	***
			poly(time,2):temperature		19.034	2.0	155.793	**4.021E-08**	***
			range:temperature		10.322	1.0	155.653	**1.598E-03**	**
			poly(time,2):range:temperature		0.228	2.0	155.876	7.962E-01	
		PHPC[3]		estimate	SE	Df	t-ratio	Pr(>t)	
			15°C: native - nonnative	8.8200	4.52000	77.0	1.952	0.0546	.
			22°C: native - nonnative	21.2900	4.85000	86.0	4.394	**< 0.0001**	***

Abbreviations: Post-hoc pairwise comparisons (PHPC), Standard error (SE), Degrees of Freedom (Df)

[1] Models assumed a Gaussian distribution and included 'population combination' and 'individual combination' as random intercepts. P-values were computed with type II Wald F-tests with Kenward-Roger Df.

[2] Tukey adjusted p-values for post-hoc timepoint comparisons were obtained using t-tests and Kenward-Roger Df, with timepoints mean estimates that were averaged over range.

[3] Tukey adjusted p-values for post-hoc range comparisons within temperature treatments were obtained using t-tests and Kenward-Roger Df.

[4] Significance codes: p < 0.1 (.), p < 0.05 (*), p < 0.01 (**), p < 0.001 (***)

Table S3. Model selection tables for alpha-diversity and within-population beta diversity.

		variable in model[1]	AICc	Df	LL	delta AICc	AICc weight
alpha-diversity	EON	poly(time,2)	941.6	15.000	-453.6	0.000	0.99817
		timepoint (factorial)	954.2	19.000	-454.5	12.600	0.00183
		time	1090.3	11.000	-533.0	148.700	0.00000
	logit PIE	**variable in model**	**AICc**	**Df**	**LL**	**delta AICc**	**AICc weight**
		poly(time,2)	253.0306	15.000	-109.4	0.000	1.00000
		timepoint (factorial)	290.2739	19.000	-122.6	37.243	0.00000
		time	345.3541	11.000	-160.5	92.324	0.00000
beta-diversity within populations	Bray-Curtis	**variable in model**	**AICc**	**Df**	**LL**	**delta AICc**	**AICc weight**
		poly(time,2)	-654.0059	15.000	343.4	0.000	1.00000
		sqrt(time)	-623.5948	11.000	323.5	30.411	0.00000
		log(time)	-613.5656	11.000	318.5	40.440	0.00000
		time	-594.512	19.000	318.5	59.494	0.00000
		timepoint (factorial)	-590.1518	11.000	306.8	63.854	0.00000
	Euclidean	**variable in model**	**AICc**	**Df**	**LL**	**delta AICc**	**AICc weight**
		poly(time,2)	1350.873	15.000	-659.0	0.000	0.99924
		time	1365.235	19.000	-661.4	14.362	0.00076
		sqrt(time)	1437.982	11.000	-707.2	87.109	0.00000
		timepoint (factorial)	1450.701	11.000	-713.6	99.828	0.00000
		log(time)	1489.877	11.000	-733.2	139.004	0.00000

Abbreviations: Effective OTU Numbers (EON), Probability of interspecific encounter (PIE), Akaike information criterion corrected for small sample sizes (AICc), degrees of Freedom (Df), log Likelihood (LL)

[1] Full models included in addition to time, the variables, temperature, range, all possible interactions and in the case of alpha-diversity the random intercepts 'population identity' and 'individual identity' and in the case of beta-diversity' population combination' and 'individual combination'.

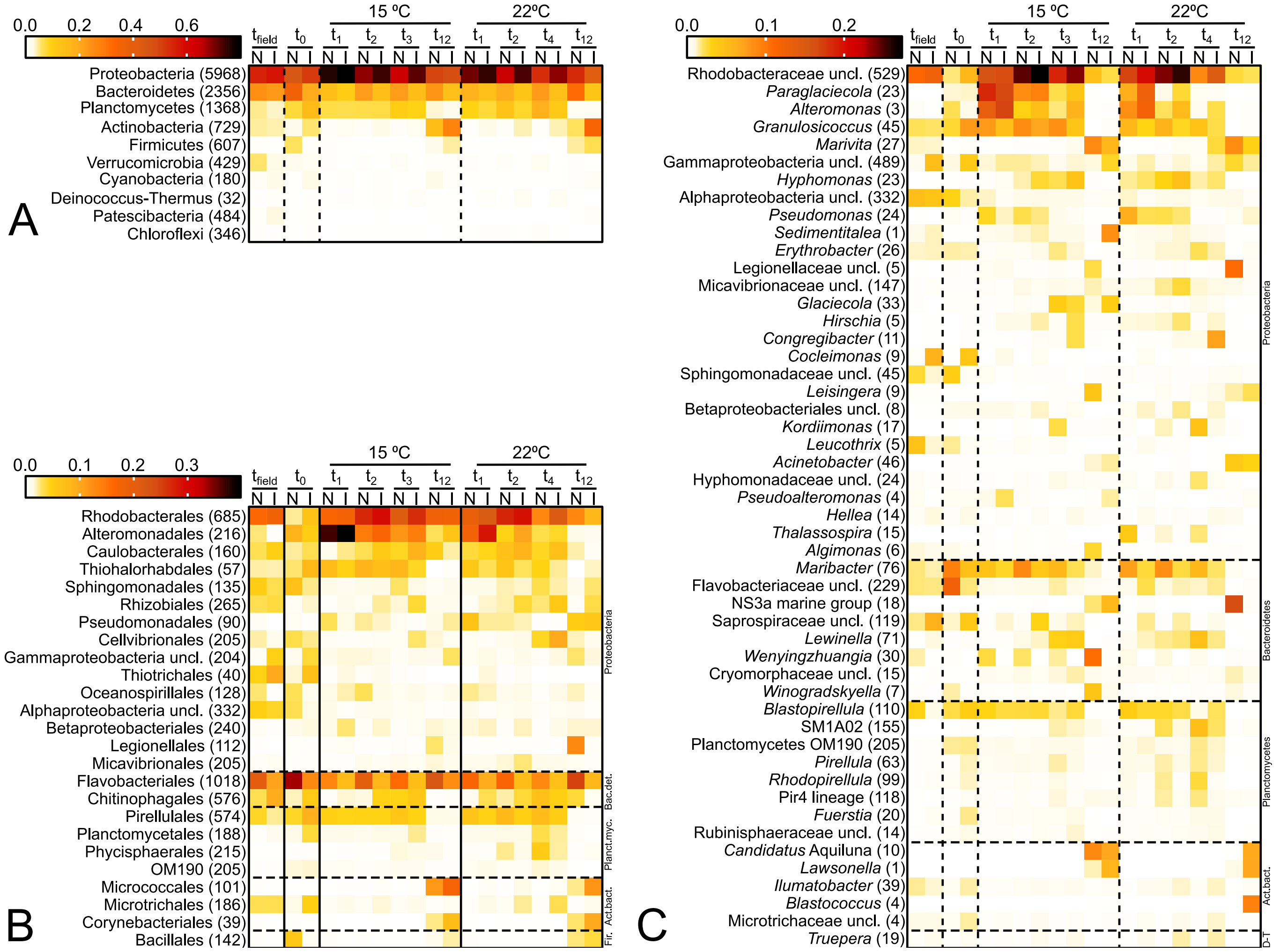

Figure S1. Heat maps reflecting the relative abundances of the 10 most abundant phyla (A), the 25 most abundant orders (B) and the 50 most abundant genera, aggregated by time (t_f, t_0, t_1, t_2, t_4 and t_{12}), temperature (15 °C and 22 °C) and range (N: native, I: introduced). The number of OTUs in each taxon is indicated between brackets behind the taxon classification.

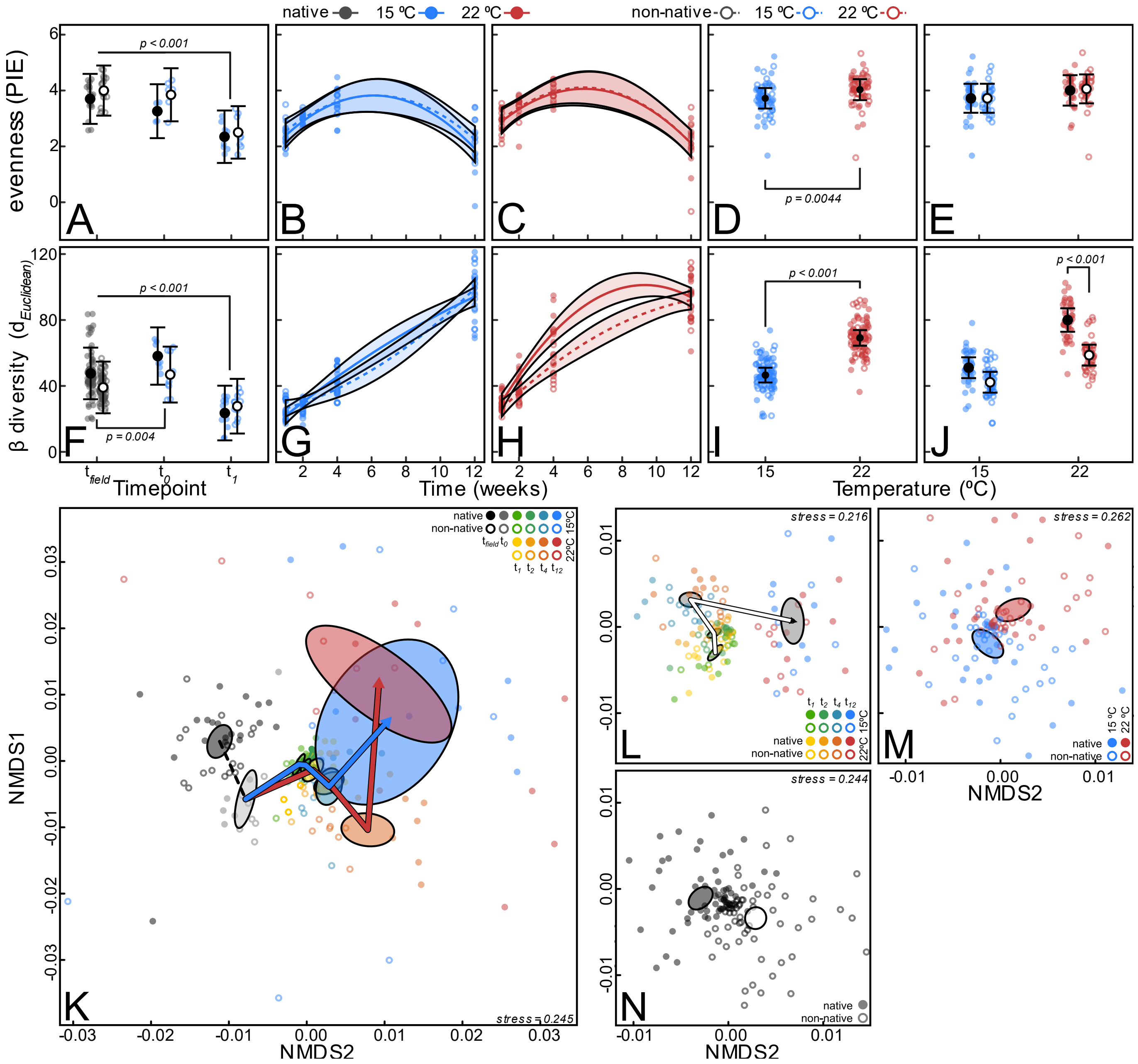

Figure S2. Evenness in terms of the logit transformed probability of interspecific encounter (PIE, A-E) and within-population beta diversity measured as Bray-Curtis distances (F-J) with 95% confidence limits and partial residuals. Marginal means in the field (t_{field}), before holobiont disturbance (t_0) and 7 days after holobiont disturbance at 15 °C (t_1, A, F). Post-disturbance responses over time at 15 °C (B, G) and 22 °C (C, H), by temperature (D, I) and by range within temperature (E, J). Non-metric dimensional scaling (NMDS) based on the rescaled residuals from mGLMs including the LSD as offset to correct for the effect of sequencing depth using Euclidean distances from pre- and post-disturbance time points (K, timepoints t_f, t_0, t_1, t_2, t_4 and t_{12}). Blue and red arrows draw the post-disturbance trajectories of the 15 and 22 °C temperature groups in time. The panels L-N show NMDS plots using rescaled residuals from mGLMs including all terms, except the variable of interest; time (B), temperature (C) and range (D). The 95% confidence regions of the group centroids are shown as ellipses. Note that some datapoints are outside the frame limits.

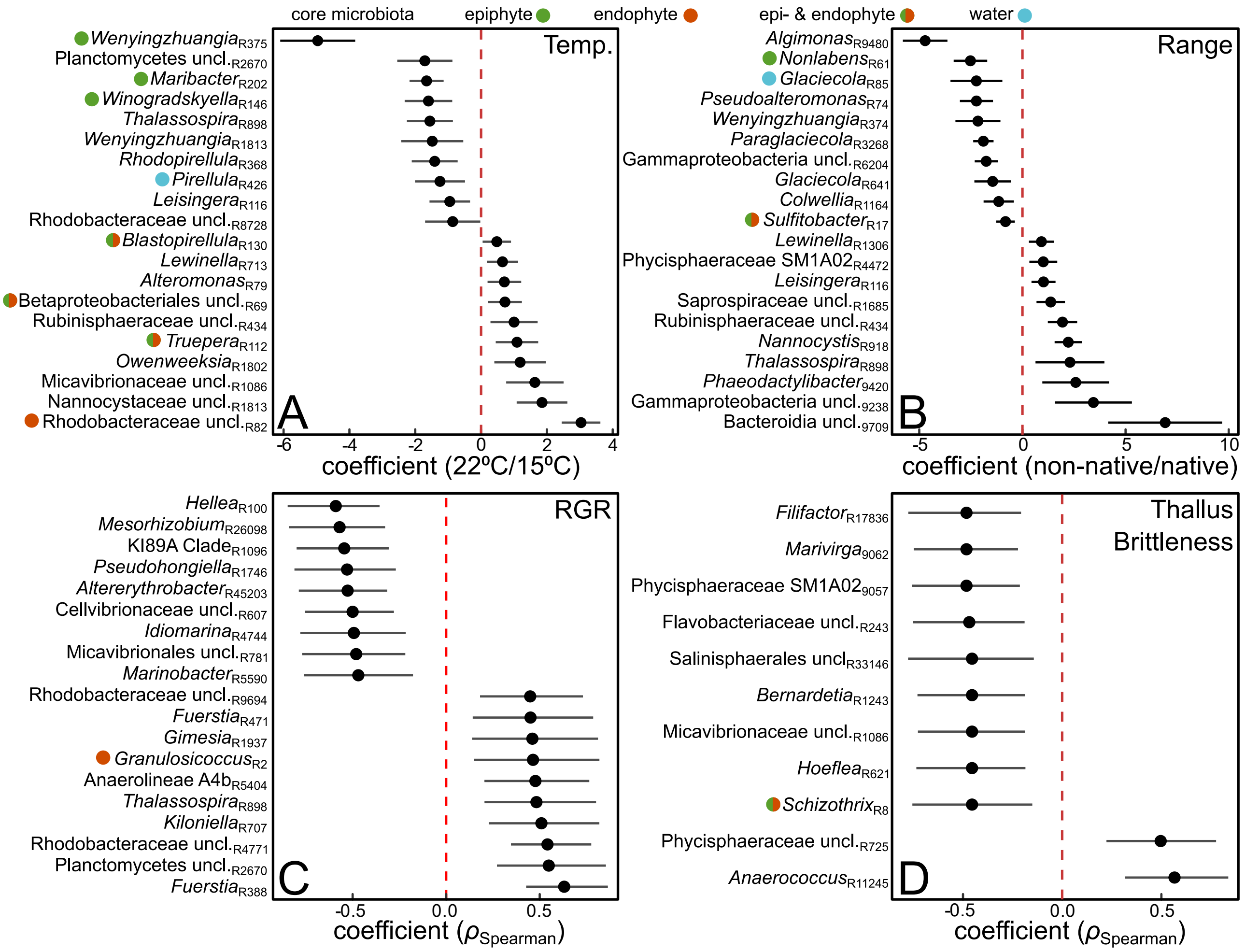

Figure S3. OTU-level analysis. (A) Model coefficients and 95% confidence intervals of the 10 most abundant OTUs significantly associated to 15 °C (blue) and the 5 most abundant OTUs significantly associated to 22 °C (red) calculated from the model comparing temperatures over the time points t_1, t_2, t_4 and t_{12}. (B) Model coefficients and 95% confidence intervals of the 10 most abundant OTUs associated with native and the 10 most abundant OTUs associated with non-native populations over the time points t_1, t_2, t_4 and t_{12}. (C) Spearman's rank correlation coefficients and 95% confidence intervals of the 10 most abundant OTUs positively associated with relative growth rate (RGR) and the 10 most abundant OTUs negatively associated with RGR. (D) Spearman's rank correlation coefficients and 95% confidence intervals OTUs positively associated with the thallus brittleness disease symptom and OTUs negatively associated with thallus brittleness. OTUs are labeled with genus classification and the OTU number in subscript. OTUs from the global field study (Bonthond et al., 2020) are labeled with an 'R', whereas OTUs without this label were only identified in the current study. OTUs that were identified as core OTUs in the same study are labeled with red, green or blue (endophyte, epiphyte or water, respectively) The confidence intervals of model derived coefficients (A, B) were calculated by multiplying the standard error with 1.96 which was summed with and subtracting from the mean. For Pearson correlation rank coefficients, confidence intervals were obtained by bootstrapping the data 1000 times.
